# Can Insect Meal Replace Fishmeal? A Meta-Analysis of the Effects of Black Soldier Fly on Fish Growth Performances and Nutritional Values

**DOI:** 10.3390/ani12131700

**Published:** 2022-06-30

**Authors:** Armel Gougbedji, Johann Detilleux, Philippe A. Lalèyè, Frédéric Francis, Rudy Caparros Megido

**Affiliations:** 1 Unit of Functional and Evolutionary Entomology, Gembloux Agro-Bio Tech, University of Liege, 5030 Gembloux, Belgium; frederic.francis@uliege.be (F.F.); r.caparros@uliege.be (R.C.M.); 2 Laboratory of Hydrobiology and Aquaculture, Faculty of Agronomics Sciences, University of Abomey-Calavi, Cotonou 01 BP 526, Benin; laleyephilippe@gmail.com; 3 Unit of Fundamental and Applied Research for Animals and Health, University of Liege, 4000 Liege, Belgium; jdetilleux@uliege.be

**Keywords:** *Hermetia illucens*, fish feed, replacement, meta-analysis, efficiency

## Abstract

**Simple Summary:**

Since 2007, black soldier fly meal has become the main substitute suggested in studies to replace fish meal in fish feeds. The quantitative results of these studies have been analyzed in this paper in order to assess the relevance of such substitution. The analysis focused on the impact of this insect on the growth and nutritional quality of fish. The results showed variable conclusions between studies. These variations are due to the fish species or to the protein substitution rate of the fish meal. Although no definite conclusions have been reached, it is possible to consider high levels of substitution.

**Abstract:**

The search for quality alternatives to fishmeal and fish oil in the fish feed industry has occupied many researchers worldwide. The use of black soldier fly meal (BSFM) as a substitute has increased. This study evaluated the effect of this substitution on fish growth and nutritional quality through a meta-analysis of the literature. A list of studies was selected after an exhaustive literature search followed by the extraction of growth and nutritional parameters. Two random-effects models were used to estimate the differences between the experimental parameters and the controls. The results showed significant heterogeneity between studies for all parameters. The sources of heterogeneity between studies were mainly fish species and protein substitution rate. High substitutions can be considered without necessarily worrying about an adverse effect. Financial profitability studies of the fish production chain from BSFM should be carried out to validate or invalidate the economic viability of this substitution.

## 1. Introduction

Aquaculture production has been growing worldwide rapidly for several decades to contribute to food security [1]. Its exponential growth is currently experiencing major economic and ecological issues as the supply of fishmeal and fish oil for fish feed formulation is constantly decreasing [2]. The increasing scarcity of these resources jeopardizes the sustainability of the wild fish fauna. It increases the costs of fish feed with a direct effect on the economic profitability of fish farming [3].

The search for adequate substitutes to fishmeal led to the belief that insects may become one of the future protein sources for animal production, principally thanks to their attributes both biological (i.e., fast reproductive and developmental cycle or ability to feed on organic residues for several species) and nutritional (i.e., high protein and fat content rich in essential fatty acids coupled with a high feed conversion efficiency) [4,5]. The most studied insect species is the dipteran *Hermetia illucens* (L.1758), commonly named “Black Soldier Fly” (BSF) [6]. Several studies have attempted to rear fish on diets based on BSF meals (BSFM) with mixed results. They analyzed several parameters in various fish species in response to partial or total substitution of fish meal (FM) by BSFM. Literature reviews have well-identified encouraging results of insect dietary inclusion on fishes’ growth and nutritional quality [7,8,9,10]. However, these classical synthesis methods based on a qualitative approach include significant subjectivity. Moreover, the sources of heterogeneity related to the experimental conditions between studies are multiple and are rarely taken into account.

The meta-analysis of data resulting from a nearly exhaustive list of studies is an aggregative method of knowledge synthesis allowing an inferential approach [11]. It is performed on quantitative data derived from different studies to provide an overall estimate of the effectiveness of an intervention and a measure of its accuracy, and is generally achieved by a Bayesian approach [12,13]. The meta-analysis performed by Hua [6] was about quantifying the effects of different insect species’ meals on the growth performance of fish. This author showed that BSFM inclusion rates below 29% did not affect fish growth and that growth decreased at higher inclusion levels. The study bases its analysis on the optimal inclusion levels of insect meal, and its approach focused on the response ratio of fish to insect meal incorporation rates. However, its methods were limited as they did not consider factors that could influence the variations between studies for the same insect species. The fish species, its ecosystem, and the experimental conditions are all factors likely to introduce heterogeneity between studies in a meta-analysis. The same author also mentioned the importance of adjusting the inclusion rate of BSFM according to the nutritional balance of the diet. Considering only the inclusion rate of BSFM in the diet can lead to a protein imbalance since protein levels in FM are generally 55–70% and BSF levels are 35–50%. Thus, the optimal limits of BSFM inclusion shown by this study may not be valid. In addition, the specific growth rate (SGR) used as the growth parameter in response to the inclusion of BSFM in the diet may not be the best evaluation option for such a study. Its limitation stems from the fact that it does not properly represent the growth trajectory of fish, which varies with developmental stages [14]. Thus, fish growth must be standardized before any comparison can be made. The best mathematical model remains the use of the thermal growth coefficient (TGC) which includes fish weight as well as temperature and rearing time in each study [14]. Furthermore, this meta-analysis in [6] did not consider the nutritional effects on fish quality. These parameters are also important in the final choice of an efficient substitute.

Therefore, the currently presented research aims to evaluate the effect of the substitution of fishmeal with BSFM meal on fish growth and nutritional value through a meta-analysis.

## 2. Materials and Methods

### 2.1. Study Search

To build the experimental meta-database, systematic bibliographic research was conducted in June 2022 in the search engine of the University of Liege (ULiège library). This library includes several other search engines: Google Scholar, Isidore, Lens, Microsoft Academic, PubMed, Scopus, Scribe and Unicat. A query formulated from keywords related to the research topic has been introduced. The syntax of the query is as follows: (“Black soldier fly”) OR (“*Hermetia illucens*”) AND Fish AND Substitution OR Replacement.

### 2.2. Selection

Once the query results were obtained, the facets (type of document, subject) of the search engine were used to reduce, step by step, the number of proposed answers to limit the noise. Scientific articles were retained after eliminating conference proceedings, patents and press articles. Articles dealing with the incorporation of BSF in poultry feed and ornamental fish such as zebrafish were excluded. The next level of sorting was to keep articles related to the use of BSF in fish feed based on the titles and abstracts. The final selection was made based on the simultaneous presence in the data of studies describing the growth, nutritional composition of BSFM, fish diets and fish reared with BSFM. The water temperature of the rearing environments was also to be provided.

### 2.3. Data Extraction

A data extraction form from each article was designed and used by two meta-analysts independently. Information collected from each manuscript was: author, year of publication, country, fish species, FM protein and lipid content (%), BSFM protein and lipid content (%), final (FBW, g/fish) and initial (IBW, g/fish) body weight, temperature (T, °C), experiment duration (D, day), feed conversion ratio (FCR), protein (Prot, %) and lipid (Lip, %) levels in fish whole-body.

The substitution levels of FM by BSFM were adjusted to the percentage of protein and lipid of FM replaced by BSFM, based on the protein content of these ingredients extracted from studies. The food habits of the fish (carnivorous or omnivorous) were assigned to each species by referring to the FishBase catalog. The TGC of each study was computed following the formula:

TGC = (FBW^1/3^ − IBW^1/3^)/∑ (T × D) × 100

The above parameters’ measures of variability (the standard deviation or standard error of the mean) were also extracted for analysis. Standard deviations not provided in the studies were generated by the multiple imputation method of missing variances [15]. For this purpose, it was assumed that each of the missing variances has a distribution equal to the true study-specific variance times a chi-square random variable divided by its degrees of freedom. It was assumed that the true variance came from a main lognormal distribution with an overall mean and precision. This cross-study distribution of true variance was estimated from studies that reported variances, and was then used to impute the variances of studies that reported an estimate of central tendency but not variance. For the particular case of TGC, which is not directly computed in studies, a simulation of standard deviations was performed from a model whose assumptions are: normally distributed weights, independent initial and final weights, fixed temperature and duration. Appendix B and Appendix C contain the code used for the imputation with the Bayesian statistical software WinBUGS-14.

### 2.4. Data Analysis

The analytical method was adapted from Moula and Detilleux [16]. For each measure, differences between means of the experimental and the control (0%) groups were computed. These differences were named DIFF_TGC, DIFF_FCR, DIFF_PROT, DIFF_LIP for TGC, FCR, Prot and Lip, respectively.

The meta-analysis was performed using two random-effects models.

Model 1: y_i_ = µ + t_i_ + e_i_

where:

y_i_ is the estimated measure (DIFF_TGC, DIFF_FCR, DIFF_PROT, DIFF_LIP) for the i_th_ trial (i = 1, 2, ..., N), N being the number of trials included in the meta-analysis, µ being the overall mean of all trials.

t_i_ and e_i_ each represent the expression of the random effects of the model. They are assumed to be independent with zero means and respectively inter-study (v^t^i) and intra-study (v^e^i) variances.

The I2 index assessed the extent of heterogeneity between studies. It measures the percentage of total inter-study variation that cannot be explained by only chance but by the number of studies analyzed [17]. For example, I^2^ values greater than 50% for the same parameter suggest heterogeneity between studies. The higher the value is, the larger the differences between studies become. Effect size estimates from each study as a function of sample size were plotted on a funnel plot. This allows detection of possible publication bias related to the studies in the meta-analysis. In case of absence of publication bias, the effects obtained will be homogeneously distributed around the true effect size. Conversely, this distribution is not homogeneous when there is a publication bias. The statistical test related to this heterogeneity is the Egger Test [18]. This models the relationship between effect sizes and their precision to determine whether the intercept of the linear regression line is null. In case of asymmetry, the intercept will not pass through zero [19].

Model 2: y_ijklmn_ = µ + t_i_ + h_ij_ + s_ik_ + c_il_ + z_im_ + q_in_ + b_1_ p_ijklmn_ + b_2_ a_ijklmn_ + e_ijklmn_

where:

y_ijkl_ is the measure for the i_th_ trial (i = 1, 2, ...., N), j_th_ (j = 1, 2, ....., 17) fish species, k_th_ fish feeding habits (k = 1, 2), l_th_ average temperature in each study, m_th_ diet protein and n_th_ diet lipid;

h_ij_, s_ik_, c_il_, z_im_ and q_in_ are the fixed effects for the j_th_ fish species, k_th_ fish feeding habits, l_th_ average temperature, m_th_ diet protein and n_th_ diet lipid, respectively. The covariate effect p_ijklmn_ is the protein fishmeal substitution rate for BSFM and covariate effect a_ijklmn_ is the lipid fishmeal substitution rate for BSFM. All these effects represent potential sources of heterogeneity between measures y_ijklmn_;

b_1_ and b_2_ are the regression coefficients linking the protein substitution rate and lipid substitution rate to the measure y_ijklmn_.

The different models were run in the R-4.02 software. The package “bayesmeta” was used to construct the funnel plots and obtain the models’ effects estimates.

## 3. Results

### 3.1. Description of Studies Included in the Meta-Analysis

**Figure 1:**
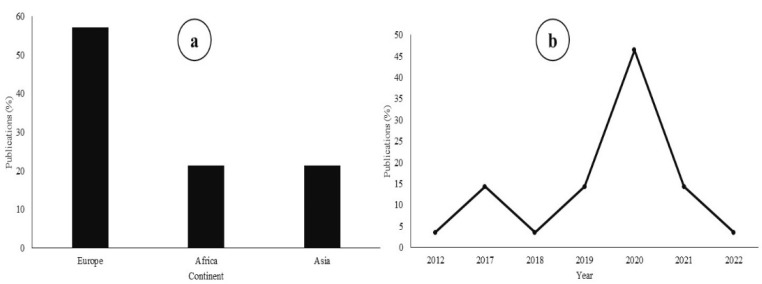
Distribution of publications according to continents (**a**) and years (**b**).

After screening the literature search through the different selection filters, 28 studies were kept for the meta-analysis (Table A1). The majority of the studies were carried out in Europe (57%) while the remaining studies were conducted in Africa (21%) and Asia (21%) (Figure 1a). Based on the criteria included in the literature search, 46% of the studies which replaced FM by BSFM were conducted in 2020 (Figure 1b).

**Figure 2:**
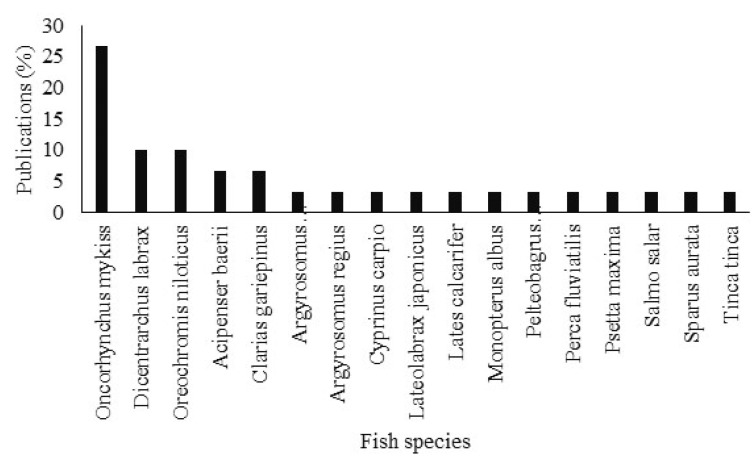
Relative abundance of fish species by publications identified in the meta-analysis.

From all studies identified, 17 species of fish including 12 carnivorous (70.59%) and 5 omnivorous (29.41%) were tested for nutrition with BSFM. The species with the highest occurrence (27%) is *Oncorhynchus mykiss* (Walbaum, 1792). Species *Dicentrarchus labrax* (Linnaeus, 1758) and *Oreochromis niloticus* (Linnaeus, 1758), each represent 10% of the publications (Figure 2).

**Figure 3:**
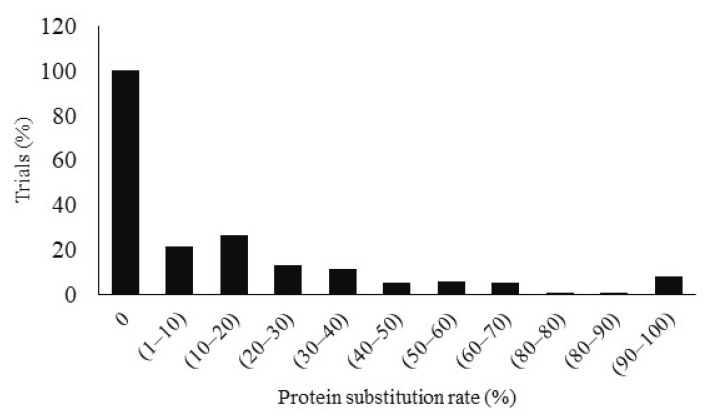
Protein substitution rate of fish meal by Black Soldier Fly meal according to trials.

The protein substitution rates of fishmeal by BSFM varied from 0 to 100% (Figure 3). Several experiments reached protein substitution rates between 10 and 20% in 26.80% of the studies. Other studies (21.65%) achieved substitution rates between 1 and 10%. Fewer studies (13.40% and 11.34%) substituted protein levels of 20–30% and 30–40% respectively. Only some studies (8.24%) replaced more than 90% of the FM protein level with BSFM. The remaining studies achieved substitution rates ranging from 40 to 90%. All experimental diets are tested against a control (0%) in all studies.

### 3.2. Search for Bias between Studies

**Figure 4:**
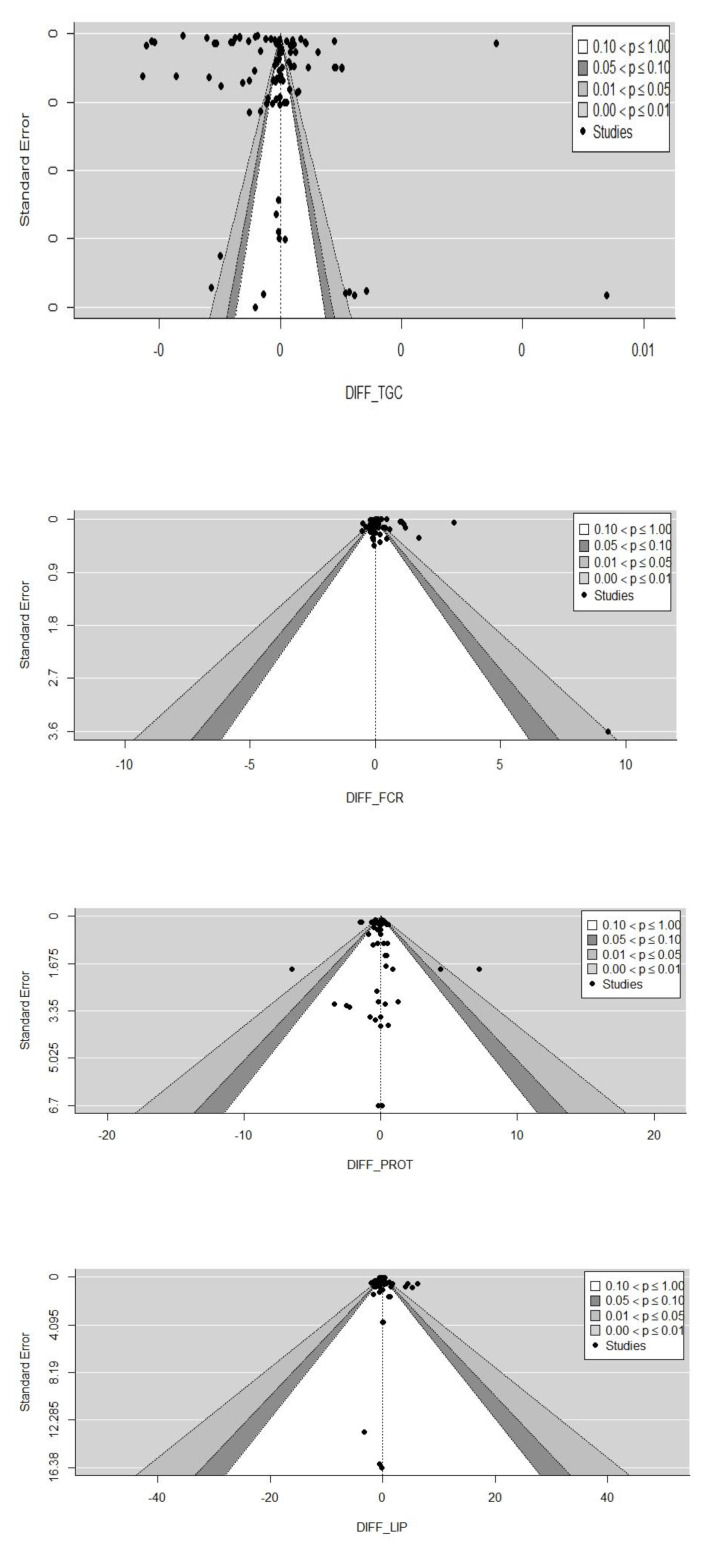
Funnel plots of differences in means between experimental and control groups.

The dispersion of effect estimates across individual studies relative to the standard error is shown for each parameter (DIFF_TGC, DIFF_FCR, DIFF_PROT, DIFF_LIP) in Figure 4. Each dot on the funnel represents one study. The effect of the substitution of FM by BSFM on one of the growth or nutritional parameters is represented on the *x*-axis. The standard error is shown on the *y* axis. Most studies cluster on the top of the funnel; this results in high precision within studies for each estimated parameter. Moreover, the studies included for these parameters mostly used large sample sizes.

For each funnel plot, the null hypothesis is rejected outside the white and dark grey areas (*p* < 0.05). This could imply that the effect of experimental diets on fish growth and nutritional parameters would not be significantly different from the control.

There was also considerable heterogeneity between studies, with I^2^ ranging from 65.13% to 99.98%.

The Egger tests (Z) performed on the biological parameters showed the presence of publication bias for DIFF_TGC (Table 1). In contrast, no publication bias was detected with DIFF_FCR, DIFF_PROT and DIFF_LIP. Except for DIFF_FCR, all represented asymmetries are negative (b < 0).

### 3.3. Sources of Heterogeneity

The potential sources of heterogeneity (Effects) between the results of the studies in the meta-analysis are presented in Table 2. These effects are assigned to the differences between the means of each parameter and their controls. The first finding is that “feeding habit” does not explain a portion of the heterogeneity between studies. This parameter was hidden in the mixed model results. For each of the other effects, they explain at least 44.41% (R2) of the variability between studies. For all parameters considered, the “temperature” effect was not significant.

Only three species seem to be the source of the heterogeneity observed for DIFF_TGC (*p* < 0.05). Fish species also had a significant effect on DIFF_FCR but was not noticeable on DIFF_PROT and DIFF_LIP. The other effects considered had no discernible impacts on fish growth. Across these studies, an increase of 1% BSFM protein has the moderate effect of increasing the overall DIFF_FCR of fish by 0.02%. In contrast, a 1% increase in lipid substitution in the feed seems to induce a decrease in the DIFF_FCR and in the level of lipid in the fish. The meta-analysis does not show an effect of the protein and lipid composition of the diets on DIFF_FCR and DIFF_PROT but shows an influence on the lipid level in the fish.

## 4. Discussion

Studies dealing with FM substitution by BSFM have increased significantly in recent years. The challenge of finding high quality alternatives to FM remains a major concern for the fisheries sector. The temporal trend of publications displayed in this study does not correctly reflect the density of studies conducted on this topic. The criteria we imposed on candidate studies in methods omitted numerous publications for which sufficient environmental or biological data was unavailable. As such, these results should not be interpreted beyond the scope we utilize them for: “the analysis of BSF inclusion in fish diets for the specific response variables selected (TGC, FCR, Prot and Lip)”. The drop in the number of studies observed in 2022 is temporary because the meta-analysis carried out only occurs in the middle of the year. Studies on the subject continue to be published. The data structure of this meta-analysis shows a dominance of studies conducted in Europe. This result can be explained by establishing a correlation with the species of fish studied. The most frequent species in the data is *O. mykiss* which is mainly reared in Europe.

The effect of FM substitution with BSFM on different fish species was analyzed in this study. Understanding the biological behavior of fish in response to a dietary change requires the integration of several growth and nutritional parameters. Four parameters were studied in this meta-analysis: TGC, FCR, protein and lipid levels in the whole body of fish. Negative asymmetries were found in the meta-analysis except for FCR, suggesting publication bias. This kind of bias generally occurs because research publications depend on the statistical significance of the results or on the direction some researchers give them [19,20]. Analysis of the specific sources of the observed variability showed that the selected effects explained more than 43.81% of the heterogeneity between studies. Other effects not considered may also include variations.

The main difficulty of a meta-analysis on fishes lies in the comparison of parameters affected by the intrinsic traits of each species. Thus, the diversity of fish makes this analysis arduous. The use of TGC as a measure of growth could limit interpretation bias. The use of the protein substitution level instead of the BSFM inclusion level could enhance the conclusions of this study. Overall, the results of this analysis suggest that high substitution levels had no effect on fish growth. This trend is in contrast to the findings of Hua’s [6] study, which suggest a decline of fish growth when BSFM inclusion rates exceed 29 ± 3% in the diet. However, the SGR used in that study as a parameter to measure growth is likely to induce an interpretation bias since it concerns different species and different stages of development. Similarly to Hua, Liland et al. [21] noted a linear decrease in SGR of fish species with an increase in BSF level in the fish diet. The effect of feeding habits was hidden in the analysis performed. Feeds for fish are usually formulated from a combination of plant and animal resources in an attempt to satisfy their nutritional requirements. Thus, the expression of their natural feeding instincts may be greatly restricted. Our results provide a more optimistic view of the incorporation of BSF into fish diets than previous studies, but the appropriate levels will also depend on a combination of several factors such as other abiotic parameters (Oxygen, Salinity, pH, dissolved solids levels etc.), fish species and nutritional details of both larvae and fish.

Other factors may also be important in the response of the fish. The fatty acid compositions of the BSF could substantially impact fish growth and fish quality. BSF are naturally rich in saturated fatty acids which limit their inclusion in the fish feed [22]. In most of the studies used in this meta-analysis, the insect meals used were not modulated to improve fatty acid quality. Several studies have already shown that the quality of BSF prepupae is greatly improved when their diets are nutritionally enriched [22,23,24,25]. Another factor that may influence digestibility and thus growth performance in fish is the high proportion of chitin in BSF prepupae [9,26].

All the trials in this study focused on the biological aspects of the use of BSF in fish feed. However, the search for substitutes for fish meal and fish oil has become urgent, particularly in view of the cost of fish production influenced by the cost of fish meal [1,8,27]. The economics of fish production from BSF therefore need to be assessed to estimate the profitability of such an initiative.

## 5. Conclusions

This study reviewed the literature on substituting fish meal with black soldier fly meal in fish diets. The quantitative method used allows for more accurate preliminary conclusions than previous studies. The issue of total substitution of FM by BSFM is not completely resolved; however, it is conceivable that high levels of substitution could be achieved without necessarily risking a negative impact on fish. In addition, economic considerations would allow future research to be directed towards ways to optimize the profitability of fish fed with BSF.

## Figures and Tables

**Table 1:** Funnel Asymmetry Tests.

Parameters	Z	*p*	b	Publication Bias
DIFF_TGC	2.73	0.01	−0.00	Yes
DIFF_FCR	0.73	0.47	0.06	No
DIFF_PROT	0.61	0.54	−0.24	No
DIFF_LIP	0.54	0.59	−0.15	No

**Table 2:** Estimation of effects responsible for sources of heterogeneity across studies; * (*p* < 0.05).

Effects		DIFF_TGC	DIFF_FCR	DIFF_PROT	DIFF_LIP
Fish species	*Acipenser baerii (reference)*	0	0	0	0
	*Argyrosomus japonicus*	0.0018 [−0.001, 0.004]	−1.30 [−2.88, 0.28]		
	*Argyrosomus regius*	−0.0005 [−0.002, 0.001]	−0.52 [−1.39, 0.35]	−2.1 [−5.29, 1.09]	−1.63 [−4.63, 1.36]
	*Cyprinus carpio*	0.0003 [−0.001, 0.002]	−0.82 [−1.65, 0.01]	−4.79 [−12.34, 2.76]	−3.63 [−7.83, 0.56]
	*Clarias gariepinus*	−0.0002 [−0.002, 0.001]	−0.45 [−1.13, 0.22]	−4.36 [−10.20, 1.49]	0.92 [−2.89, 4.74]
	*Dicentrarchus labrax*	0.0002 [−0.001, 0.001]	−0.44 [−1.14, 0.26]	−1.37 [−3.69, 0.96]	0.39 [−2.47, 3.26]
	*Lates calcarifer*	−0.0003 [−0.002, 0.001]	0.17 [−0.50, 0.85]	−1.72 [−6.71, 3.26]	0.02 [−3.08, 3.13]
	*Lateolabrax japonicus*	0.0006 [−0.001, 0.002]	−0.38 [−1.36, 0.61]	−4.59 [−10.50, 1.31]	−4.89 [−8.77, −1.01]
	*Monopterus albus*	0.0002 [−0.001, 0.002]	−0.64 [−1.38, 0.09]	−5.19 [−11.41, 1.03]	−1.73 [−19.21, 15.75]
	*Oncorhynchus mykiss*	0.0000 [−0.001, 0.001]	0.14 [−0.58, 0.86]	0.30 [−2.08, 2.67]	−1.76 [−4.50, 0.98]
	*Oreochromis niloticus*	0.0003 [−0.001, 0.002]	−0.71 [−1.52, 0.11]	−5.17 [−11.96, 1.62]	−0.09 [−4.32, 4.14]
	*Perca fluviatilis*	−0.0007 [−0.002, 0.000]	−0.02 [−0.58, 0.55]	−6.02 [−11.25, −0.79]	4.49 [1.01, 7.97]
	*Pelteobagrus fulvidraco*	0.0016 [0.000, 0.003] *	−0.91 [−1.76, −0.05] *	−4.50 [−9.93, 0.92]	−1.15 [−5.01, 2.72]
	*Psetta maxima*	−0.0018 [−0.003, −0.001] *	0.18 [−0.28, 0.63]	−1.27 [−4.83, 2.28]	−1.35 [−0.53, 3.22]
	*Sparus aurata*	−0.0009 [−0.002, 0.001]	0.1 [−0.68, 0.87]		
	*Salmo salar*	−0.0002 [−0.002, 0.001]	0.18 [−0.57, 0.94]	−0.52 [−2.80, 1.75]	−2.08 [−4.42, 0.26]
	*Tinca tinca*	−0.0022 [−0.004, −0.001] *	1.01 [0.23, 1.79] *		
Protein substitution rate		−0.0000 [0.0000, 0000]	0.02 [0.01, 0.03] *	−0.01 [−0.02, 0.01]	0.07 [0.04, 0.10]
Lipid substitution rate		−0.0000 [0.0000, 0000]	−0.01[−0.02, 0.00] *	0.00 [−0.01, 0.02]	−0.09 [−0.12, −0.05] *
Diet protein		0.0001 [0.0000, 0.0001]	−0.02 [−0.07, 0.02]	0.02 [−0.10, 0.15]	−0.50 [−0.65, −0.35] *
Diet lipid		−0.0001 [−0.0002, 0.0001]	0.03 [−0.05, 0.11]	−0.18 [−0.40, 0.05]	0.42 [0.11, 072] *
Temperature		0.0000 [−0.0001, 0.0001]	0.04 [−0.01, 0.09]	0.28 [−0.05, 0.61]	−0.18 [−0.45, 0.09]
Overall mean		0.0009 [0.0008, 0.9676]	0.20 [0.11, 0.30]	0.42[0.28, 0.58]	1.53 [1.20, 1.91]
Amount of heterogeneity accounted for (R2, %)		43.81	44.41	96.38	89.57

## Data Availability

The data presented in this study are available on request from the corresponding author A.G.

## Appendix A

**Table A1:** Full list of studies used for the meta-analysis; na = missing data.

Author	Year	Country	Fish Species	SubProt	Temp	TGC		Feed Conversion Ratio		Fish Protein (%)		Fish Lipid (%)	
	2012					Mean	SD	Mean	SD	Mean	SD	Mean	SD
Kroeckel et al. [28]	2012	Germany	*Psetta maxima*	0	16.5	0.0035	0.000065	0.76	0.00	15.20	2.20	5.80	0.30
Kroeckel et al. [28]	2012	Germany	*Psetta maxima*	13.2	16.5	0.0030	0.000035	0.76	0.00	15.20	2.80	4.80	0.60
Kroeckel et al. [28]	2012	Germany	*Psetta maxima*	26.78	16.5	0.0028	0.000044	0.82	0.00	15.50	2.20	4.80	0.30
Kroeckel et al. [28]	2012	Germany	*Psetta maxima*	41.64	16.5	0.0023	0.000026	0.86	0.00	14.9	1.50	4.50	0.50
Kroeckel et al. [28]	2012	Germany	*Psetta maxima*	56.9	16.5	0.0017	0.000019	0.98	0.00	15.00	2.10	4.10	0.40
Kroeckel et al., 2012 [28]	2012	Germany	*Psetta maxima*	70.16	16.5	0.0012	0.000019	1.21	0.00	15.20	3.20	3.80	0.40
Katya et al. [24]	2017	Malaysia	*Lates calcarifer*	0	24	0.0016	0.000005	2.00	0.10	62.2	1.32	16.3	0.60
Katya et al. [24]	2017	Malaysia	*Lates calcarifer*	17.79	24	0.0014	0.000007	2.30	0.10	63.1	1.32	20.3	0.60
Katya et al. [24]	2017	Malaysia	*Lates calcarifer*	39.37	24	0.0013	0.000006	2.40	0.10	55.7	1.32	14.8	0.60
Katya et al. [24]	2017	Malaysia	*Lates calcarifer*	66.08	24	0.0012	0.000001	3.20	0.10	69.4	1.32	15.1	0.60
Katya et al. [24]	2017	Malaysia	*Lates calcarifer*	100	24	0.0004	0.000005	11.30	3.60	66.6	1.32	17.7	0.60
Magalhães et al. [25]	2017	Portugal	*Dicentrarchus labrax*	0	25	0.0033	0.000065	na	Na	na	na	na	na
Magalhães et al. [25]	2017	Portugal	*Dicentrarchus labrax*	11.45	25	0.0035	0.000067	na	Na	na	na	na	na
Magalhães et al. [25]	2017	Portugal	*Dicentrarchus labrax*	23.9	25	0.0036	0.000066	na	na	na	na	na	na
Magalhães et al. [25]	2017	Portugal	*Dicentrarchus labrax*	37.48	25	0.0034	0.000062	na	na	na	na	na	na
Renna et al. [29]	2017	Italy	*Oncorhynchus mykiss*	0	13	0.0063	0.000234	0.90	0.02	19.58	0.35	4.18	1.20
Renna et al. [29]	2017	Italy	*Oncorhynchus mykiss*	21.66	13	0.0064	0.000230	0.88	0.02	19.37	0.35	5.19	1.20
Renna et al. [29]	2017	Italy	*Oncorhynchus mykiss*	45.35	13	0.0063	0.000228	0.90	0.02	19.56	0.35	5.48	1.20
Devic et al. [23]	2018	Ghana	*Oreochromis niloticus*	0	28.65	0.0019	0.000007	2.20	0.10	15.36	0.30	10.78	0.61
Devic et al. [23]	2018	Ghana	*Oreochromis niloticus*	21.03	28.65	0.0021	0.000015	2.10	0.30	15.27	0.13	9.61	0.11
Devic et al. [23]	2018	Ghana	*Oreochromis niloticus*	38.33	28.65	0.0019	0.000011	2.00	0.20	15.29	0.09	9.99	0.44
Devic et al. [23]	2018	Ghana	*Oreochromis niloticus*	71.31	28.65	0.0018	0.000008	2.10	0.10	15.43	0.05	10.22	0.61
Xiao et al. [26]	2018	China	*Pelteobagrus fulvidraco*	0	28	0.0089	0.000294	1.08	0.07	14.3	0.1	5.59	0.08
Xiao et al. [26]	2018	China	*Pelteobagrus fulvidraco*	13	28	0.0102	0.000297	0.90	0.04	13.9	0.1	5.37	0.01
Xiao et al. [26]	2018	China	*Pelteobagrus fulvidraco*	25	28	0.0104	0.000287	0.89	0.03	14.6	0.2	5.41	0.09
Xiao et al. [26]	2018	China	*Pelteobagrus fulvidraco*	37	28	0.0101	0.000290	0.91	0.02	13.8	0.4	5.07	0.01
Xiao et al. [26]	2018	China	*Pelteobagrus fulvidraco*	48	28	0.0100	0.000293	0.93	0.04	13.7	0.2	5.22	0.10
Xiao et al. [26]	2018	China	*Pelteobagrus fulvidraco*	68	28	0.0087	0.000294	1.08	0.09	13.6	0.2	5.3	0.13
Xiao et al. [26]	2018	China	*Pelteobagrus fulvidraco*	85	28	0.0078	0.000279	1.19	0.05	12.9	0.2	5.47	0.01
Xiao et al. [26]	2018	China	*Pelteobagrus fulvidraco*	100	28	0.0054	0.000298	1.66	0.16	12.8	0.2	5.45	0.06
Cardinaletti et al. [27]	2019	Italy	*Oncorhynchus mykiss*	0	12.8	0.0039	0.000287	1.02	0.17	na	na	na	na
Cardinaletti et al. [27]	2019	Italy	*Oncorhynchus mykiss*	13.84	12.8	0.0035	0.000329	1.22	0.35	na	na	na	na
Cardinaletti et al. [27]	2019	Italy	*Oncorhynchus mykiss*	32.52	12.8	0.0029	0.000209	1.47	0.28	na	na	na	na
Józefiak et al. [30]	2019	Poland	*Oncorhynchus mykiss*	0	13.85	0.0044	0.000053	0.95	0.02	na	na	na	na
Józefiak et al. [30]	2019	Poland	*Oncorhynchus mykiss*	12.3	13.85	0.0044	0.000052	0.97	0.02	na	na	na	na
Terova et al. [31]	2019	Italy	*Oncorhynchus mykiss*	0	13	0.0050	0.000190	0.9	0.02	na	na	na	na
Terova et al. [31]	2019	Italy	*Oncorhynchus mykiss*	7.59	13	0.0050	0.000255	0.93	0.04	na	na	na	na
Terova et al. [31]	2019	Italy	*Oncorhynchus mykiss*	15.59	13	0.0050	0.000217	0.95	0.03	na	na	na	na
Terova et al. [31]	2019	Italy	*Oncorhynchus mykiss*	24.05	13	0.0050	0.000187	0.93	0.04	na	na	na	na
Wang et al. [32]	2019	China	*Lateolabrax japonicus*	0	27.4	0.0036	0.000008	1.37	0.03	17.2	0.13	8.66	0.14
Wang et al. [32]	2019	China	*Lateolabrax japonicus*	13.61	27.4	0.0037	0.000022	1.44	0.07	17.22	0.15	8.26	0.18
Wang et al. [32]	2019	China	*Lateolabrax japonicus*	28.01	27.4	0.0036	0.000018	1.41	0.05	17.13	0.18	8.25	0.38
Wang et al. [32]	2019	China	*Lateolabrax japonicus*	43.29	27.4	0.0038	0.000006	1.40	0.02	16.82	0.13	8.88	0.26
Wang et al. [32]	2019	China	*Lateolabrax japonicus*	59.51	27.4	0.0036	0.000007	4.50	0.04	16.89	0.16	8.9	0.22
Abdel-Tawwab et al. [33]	2020	Egypt	*Dicentrarchus labrax*	0	27.85	0.0043	0.000021	1.42	0.09	17.76	4.74	6.13	0.80
Abdel-Tawwab et al. [33]	2020	Egypt	*Dicentrarchus labrax*	17.68	27.85	0.0043	0.000021	1.41	0.09	17.84	4.74	6.11	0.80
Abdel-Tawwab et al. [33]	2020	Egypt	*Dicentrarchus labrax*	25.76	27.85	0.0043	0.000021	1.44	0.09	17.6	4.74	6.22	3.80
Abdel-Tawwab et al. [33]	2020	Egypt	*Dicentrarchus labrax*	39.18	27.85	0.0043	0.000022	1.42	0.09	17.57	4.74	6.13	3.80
Caimi et al. [34]	2020	Italy	*Acipenser baerii*	0	13	0.0064	0.000087	1.03	0.03	13.66	0.99	4.5	0.39
Caimi et al. [34]	2020	Italy	*Acipenser baerii*	24.62	13	0.0060	0.000089	1.08	0.03	14.1	0.99	5.13	0.39
Caimi et al. [34]	2020	Italy	*Acipenser baerii*	49.49	13	0.0058	0.000091	1.12	0.03	13.96	0.99	6.23	0.39
Fawole et al. [35]	2020	Nigeria	*Clarias gariepinus*	0	26.61	0.0022	0.000021	1.86	0.09	16.82	0.67	5.3	0.42
Fawole et al. [35]	2020	Nigeria	*Clarias gariepinus*	17.49	26.61	0.0024	0.000021	1.78	0.09	17.03	0.67	5.66	0.42
Fawole et al. [35]	2020	Nigeria	*Clarias gariepinus*	38.87	26.61	0.0028	0.000021	1.48	0.09	17.30	0.67	4.76	0.42
Fawole et al. [35]	2020	Nigeria	*Clarias gariepinus*	65.61	26.61	0.0024	0.000021	1.65	0.09	16.59	0.67	5.05	0.42
Guerreiro et al. [36]	2020	Portugal	*Argyrosomus regius*	0	22.4	0.0043	0.000075	1.25	0.03	16.80	0.16	5.97	0.27
Guerreiro et al. [36]	2020	Portugal	*Argyrosomus regius*	7.76	22.4	0.0042	0.000013	1.22	0.04	16.70	0.47	5.57	0.12
Guerreiro et al. [36]	2020	Portugal	*Argyrosomus regius*	15.91	22.4	0.0038	0.000011	1.17	0.04	16.70	0.19	5.28	0.40
Guerreiro et al. [36]	2020	Portugal	*Argyrosomus regius*	24.49	22.4	0.0033	0.000038	1.05	0.17	16.80	0.62	6.15	0.60
Hu et al. [37]	2020	China	*Monopterus albus*	0	28	0.0021	0.000007	2.04	0.01	17.25	0.12	16.6	0.14
Hu et al. [37]	2020	China	*Monopterus albus*	1.2	28	0.0025	0.000012	1.54	0.07	17.36	0.01	16.38	16.38
Hu et al. [37]	2020	China	*Monopterus albus*	2.44	28	0.0024	0.000004	1.77	0.15	17.48	0.10	16.06	16.06
Hu et al. [37]	2020	China	*Monopterus albus*	3.71	28	0.0022	0.000009	1.86	0.09	17.27	0.03	13.3	13.3
Mastoraki et al. [38]	2020	Greece	*Dicentrarchus labrax*	0	19.3	0.0028	0.000003	0.99	0.02	17.79	0.16	13.65	0.43
Mastoraki et al. [38]	2020	Greece	*Dicentrarchus labrax*	29.09	19.3	0.0037	0.000011	1.03	0.01	17.86	0.01	11.91	0.10
Xu et al. [39]	2020	China	*Cyprinus carpio*	0	27.5	0.0044	0.000025	1.22	0.06	20.43	3.03	7.20	0.33
Xu et al. [39]	2020	China	*Cyprinus carpio*	15.01	27.5	0.0044	0.000030	1.24	0.07	20.05	2.09	5.61	0.39
Xu et al. [39]	2020	China	*Cyprinus carpio*	34.63	27.5	0.0044	0.000022	1.26	0.06	21.70	0.04	5.70	0.77
Xu et al. [39]	2020	China	*Cyprinus carpio*	61.38	27.5	0.0043	0.000035	1.33	0.07	19.63	1.90	5.81	0.00
Xu et al. [39]	2020	China	*Cyprinus carpio*	100	27.5	0.0043	0.000044	1.24	0.1	21.00	2.40	5.79	0.54
Fabrikov et al. [40]	2020	Spain	*Oncorhynchus mykiss*	0	20	0.0035	0.000012	0.77	0	na	na	na	na
Fabrikov et al. [40]	2020	Spain	*Oncorhynchus mykiss*	9.55	20	0.0035	0.000014	0.78	0.01	na	na	na	na
Fabrikov et al. [40]	2020	Spain	*Oncorhynchus mykiss*	20.41	20	0.0034	0.000008	0.78	0.01	na	na	na	na
Fabrikov et al. [40]	2020	Spain	*Tinca tinca*	9.55	20	0.0014	0.000003	1.82	0.04	na	na	na	na
Fabrikov et al. [40]	2020	Spain	*Tinca tinca*	20.41	20	0.0013	0.000013	1.90	0.08	na	na	na	na
Fabrikov et al. [40]	2020	Spain	*Tinca tinca*	9.55	20	0.0014	0.000007	1.77	0.04	na	na	na	na
Fabrikov et al. [40]	2020	Spain	*Sparus aurata*	20.41	20	0.0027	0.000004	1.02	0.00	na	na	na	na
Fabrikov et al. [40]	2020	Spain	*Sparus aurata*	9.55	20	0.0027	0.000007	0.98	0.01	na	na	na	na
Fabrikov et al. [40]	2020	Spain	*Sparus aurata*	20.41	20	0.0025	0.000008	0.92	0.12	na	na	na	na
Rawski et al. [41]	2020	Poland	*Acipenser baerii*	0	20.3	0.0049	0.000039	0.88	0.01	na	na	na	na
Rawski et al. [41]	2020	Poland	*Acipenser baerii*	2.89	20.3	0.0054	0.000037	0.79	0.01	na	na	na	na
Rawski et al. [41]	2020	Poland	*Acipenser baerii*	6.61	20.3	0.0058	0.000038	0.89	0.01	na	na	na	na
Rawski et al. [41]	2020	Poland	*Acipenser baerii*	9.09	20.3	0.0058	0.000038	0.7	0.01	na	na	na	na
Rawski et al. [41]	2020	Poland	*Acipenser baerii*	12.4	20.3	0.0059	0.000039	0.68	0.01	na	na	na	na
Rawski et al. [41]	2020	Poland	*Acipenser baerii*	15.88	20.3	0.0058	0.000039	0.68	0.01	na	na	na	na
Rawski et al. [41]	2020	Poland	*Acipenser baerii*	19.53	20.3	0.0059	0.000040	0.68	0.01	na	na	na	na
Madibana et al. [42]	2020	South africa	*Argyrosomus japonicus*	0	25	0.0015	0.000011	1.73	0.14	na	na	na	na
Madibana et al. [42]	2020	South africa	*Argyrosomus japonicus*	5.92	25	0.0004	0.000011	1.20	0.14	na	na	na	na
Madibana et al. [42]	2020	South africa	*Argyrosomus japonicus*	11.73	25	0.0020	0.000012	1.20	0.14	na	na	na	na
Madibana et al. [42]	2020	South africa	*Argyrosomus japonicus*	23.02	25	0.0051	0.000012	1.66	0.14	na	na	na	na
Melenchón et al. [43]	2020	Spain	*Oncorhynchus mykiss*	0	15	0.0035	0.000053	0.77	0.02	18.61	0.2	1.28	0.04
Melenchón et al. [43]	2020	Spain	*Oncorhynchus mykiss*	7.32	15	0.0035	0.000052	0.78	0.02	19.16	0.2	1.66	0.04
Melenchón et al. [43]	2020	Spain	*Oncorhynchus mykiss*	16.1	15	0.0034	0.000054	0.78	0.02	19.06	0.2	1.27	0.04
Adeoye et al. [44]	2020	Nigeria	*Clarias gariepinus*	0	30.34	0.0025	0.000002	1.22	0.10	na	na	na	na
Adeoye et al. [44]	2020	Nigeria	*Clarias gariepinus*	16.47	30.34	0.0020	0.000011	1.41	0.24	na	na	na	na
Adeoye et al. [44]	2020	Nigeria	*Clarias gariepinus*	37.17	30.34	0.0022	0.000002	1.29	0.05	na	na	na	na
Adeoye et al. [44]	2020	Nigeria	*Clarias gariepinus*	100	30.34	0.0009	0.000003	2.96	0.30	na	na	na	na
Stejskal et al. [45]	2020	Czech Republic	*Perca fluviatilis*	0	22.5	0.0030	0.000010	1.00	0.07	24.10	3.10	10.10	1.30
Stejskal et al. [45]	2020	Czech Republic	*Perca fluviatilis*	17.18	22.5	0.0032	0.000017	0.91	0.05	21.80	0.90	9.50	0.20
Stejskal et al. [45]	2020	Czech Republic	*Perca fluviatilis*	35.61	22.5	0.0032	0.000015	0.91	0.04	21.60	0.60	8.70	0.50
Stejskal et al. [45]	2020	Czech Republic	*Perca fluviatilis*	55.45	22.5	0.0027	0.000026	1.12	0.06	20.70	0.30	8.50	0.80
Weththasinghe et al. [46]	2021	Poland	*Salmo salar*	0	14.8	0.0033	0.000071	0.77	0.07	8.72	0.17	9.52	0.11
Weththasinghe et al. [46]	2021	Poland	*Salmo salar*	6.25	14.8	0.0032	0.000076	0.78	0.07	8.69	0.17	9.46	0.11
Weththasinghe et al. [46]	2021	Poland	*Salmo salar*	12.5	14.8	0.0033	0.000072	0.76	0.07	8.47	0.17	9.13	0.11
Weththasinghe et al. [46]	2021	Poland	*Salmo salar*	25	14.8	0.0031	0.000075	0.81	0.07	8.33	0.17	8.80	0.11
Tippayadara et al. [47]	2021	Thailand	*Oreochromis niloticus*	0	28.93	0.0027	0.000034	2.22	0.17	na	na	na	na
Tippayadara et al. [47]	2021	Thailand	*Oreochromis niloticus*	4.15	28.93	0.0027	0.000049	2.15	0.27	na	na	na	na
Tippayadara et al. [47]	2021	Thailand	*Oreochromis niloticus*	8.88	28.93	0.0029	0.000029	2.15	0.10	na	na	na	na
Tippayadara et al. [47]	2021	Thailand	*Oreochromis niloticus*	20.63	28.93	0.0030	0.000040	2.14	0.31	na	na	na	na
Tippayadara et al. [47]	2021	Thailand	*Oreochromis niloticus*	36.9	28.93	0.0029	0.000041	2.16	0.42	na	na	na	na
Tippayadara et al. [47]	2021	Thailand	*Oreochromis niloticus*	60.93	28.93	0.0028	0.000043	2.16	0.17	na	na	na	na
Tippayadara et al. [47]	2021	Thailand	*Oreochromis niloticus*	100	28.93	0.0027	0.000031	2.23	0.15	na	na	na	na
Hoc et al. [48]	2021	Belgium	*Oncorhynchus mykiss*	0	12	0.0039	0.000049	1.12	0.00	39.23	0.13	14.74	0.33
Hoc et al. [48]	2021	Belgium	*Oncorhynchus mykiss*	57.44	12	0.0039	0.000045	1.23	0.03	38.78	0.18	15.97	0.27
Hoc et al. [48]	2021	Belgium	*Oncorhynchus mykiss*	58.97	12	0.0038	0.000053	1.24	0.04	38.90	0.13	15.17	0.46
Caimi et al. [49]	2022	Italy	*Oncorhynchus mykiss*	0	13	0.0048	0.000078	1.08	0.06	na	na	na	na
Caimi et al. [49]	2022	Italy	*Oncorhynchus mykiss*	2.54	13	0.0048	0.000077	1.09	0.06	na	na	na	na
Caimi et al. [49]	2022	Italy	*Oncorhynchus mykiss*	5.1	13	0.0049	0.000079	1.09	0.06	na	na	na	na
Caimi et al. [49]	2022	Italy	*Oncorhynchus mykiss*	7.68	13	0.0048	0.000082	1.12	0.06	na	na	na	na
Caimi et al. [49]	2022	Italy	*Oncorhynchus mykiss*	10.3	13	0.0046	0.000079	1.13	0.06	na	na	na	na
Caimi et al. [49]	2022	Italy	*Oncorhynchus mykiss*	12.93	13	0.0045	0.000080	1.18	0.06	na	na	na	na
Agbohessou et al. [50]	2022	Benin	*Oreochromis niloticus*	0	28.74	0.0042	0.000005	na	na	57.3	0.5	21.26	0.57
Agbohessou et al. [50]	2022	Benin	*Oreochromis niloticus*	100	28.74	0.0035	0.000004	na	na	57.69	1.7	26.46	0.74
Agbohessou et al. [50]	2022	Benin	*Oreochromis niloticus*	100	28.74	0.0034	0.000004	na	na	56.76	0.89	27.54	0.19
Agbohessou et al. [50]	2022	Benin	*Oreochromis niloticus*	100	28.74	0.0035	0.000002	na	na	56.42	0.4	25.67	0.17

## Appendix B

Example of an imputation of missing variance for continuous data

# nstudies = total number of studies (with and without missing information)

model

{

for (i in 1: nstudies)

{

trueVar[i]~dlnorm(tmu, tau)

shape[i]<-(num[i]-1)/2

scale[i]<-(num[i]-1)/(2*trueVar[i])

obsVar[i]~dgamma(shape[i], scale[i])

}

tmu~dnorm (0,0.001)

tau<-1/(sd×sd)

sd~dunif (0.01,1)

}

INITS

list (tmu=0.01, sd=0.12)

DATA

list(nstudies=31 ,num=c(60,30,30,120,90,4500,60,60,30,120,5748,60,120,90,270,270,90,390,60,120,87,90,60,88,45,120,54,150,90,60,54),obsVar=c(NA,NA,0.01,NA,NA,0.04,0.004,0.003,NA,NA,0.003,0.004,NA,0.02,0.03,NA,0.01,0.002,0.03,NA,0.0001,0.0004,0.03,0.02,0.04,0.03,NA,NA,0.001,0.0004,0.005))

## Appendix C

Example of standard deviation simulation for each TGC

#Pmi

mu1<-55

sd1<-1

X1<- rnorm(1000, mean = mu1, sd = sd1)

Y1<-X1^1/3

postmu1<-mean(Y1)

postsd1<-sd(Y1)

#Pmf

mu2<-139

sd2<-8

X2<- rnorm(1000, mean = mu2, sd = sd2)

Y2<-X2^1/3

postmu2<-mean(Y2)

postsd2<-sd(Y2)

∑TXD=3000

var1<- postsd1* postsd1

var2<- postsd2* postsd2

TXD2=TXD*TXD

mean<-(1/TXD)*(postmu2-postmu1)

var<-(1/(TXD2))*(var2+var1)

sd<-sqrt(var)

sd
